# Is the Image Quality of I-124-PET Impaired by an Automatic Correction of Prompt Gammas?

**DOI:** 10.1371/journal.pone.0071729

**Published:** 2013-08-27

**Authors:** Veronika Preylowski, Susanne Schlögl, Frédéric Schoenahl, Gerhard Jörg, Samuel Samnick, Andreas K. Buck, Michael Lassmann

**Affiliations:** 1 Department of Nuclear Medicine, University Würzburg, Würzburg, Germany; 2 Martin-Luther University Halle-Wittenberg, Halle-Wittenberg, Germany; 3 Siemens AG Healthcare, Zürich, Switzerland; NIH, United States of America

## Abstract

**Objectives:**

The aim of this study is to evaluate the quality of I-124 PET images with and without prompt gamma compensation (PGC) by comparing the recovery coefficients (RC), the signal to noise ratios (SNR) and the contrast to F-18 and Ga-68. Furthermore, the influence of the PGC on the quantification and image quality is evaluated.

**Methods:**

For measuring the image quality the NEMA NU2-2001 PET/SPECT-Phantom was used containing 6 spheres with a diameter between 10 mm and 37 mm placed in water with different levels of background activity. Each sphere was filled with the same activity concentration measured by an independently cross-calibrated dose calibrator. The “hot” sources were acquired with a full 3D PET/CT (Biograph mCT®, Siemens Medical USA). Acquisition times were 2 min for F-18 and Ga-68, and 10 min for I-124. For reconstruction an OSEM algorithm was applied. For I-124 the images were reconstructed with and without PGC. For the calculation of the RCs the activity concentrations in each sphere were determined; in addition, the influence of the background correction was studied.

**Results:**

The RCs of Ga-68 are the smallest (79%). I-124 reaches similar RCs (87% with PGC, 84% without PGC) as F-18 (84%). showing that the quantification of I-124 images is similar to F-18 and slightly better than Ga-68. With background activity the contrast of the I-124 PGC images is similar to Ga-68 and F-18 scans. There was lower background activity in the I-124 images without PGC, which probably originates from an overcorrection of the scatter contribution. Consequently, the contrast without PGC was much higher than with PGC. As a consequence PGC should be used for I-124.

**Conclusions:**

For I-124 there is only a slight influence on the quantification depending on the use of the PGC. However, there are considerable differences with respect to I-124 image quality.

## Introduction

Frequently, radioiodine therapy is the treatment of choice in patients suffering from differentiated thyroid cancer (DTC) [1]. It is defined as the systemic administration of I-131 sodium or potassium iodide for selective irradiation of thyroid remnants, microscopic DTC or other non-resectable or incompletely resectable DTC, or both purposes [1]. The determination of the I-131 biokinetics of iodine-avid lesions for individualized treatment planning suffers from severe shortcomings of the quantification process using gamma-cameras mainly because of the high I-131 photon energies [2]. Therefore, sequential PET(/CT)-measurements with I-124 represent a suitable substitute because of the same pharmacokinetics as I-131. With the long half-life of I-124 of 4.17 d, slow metabolic processes can be examined which cannot be assessed with I-123 (half-life 13.2 h) which is used for conventional scintigraphy [3]–[5].

**Figure 1:**
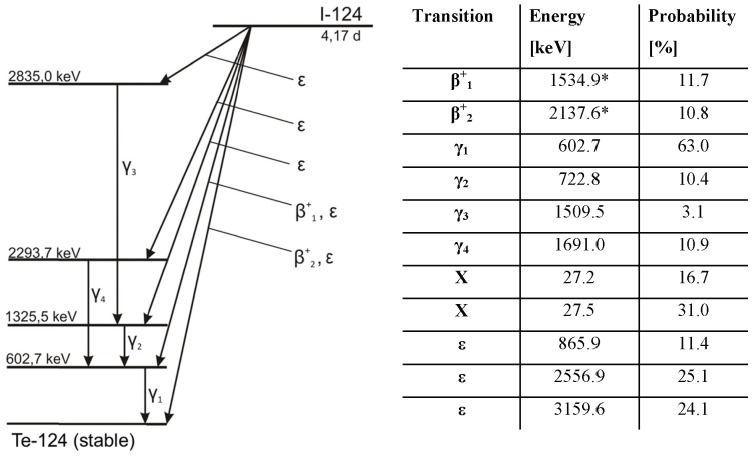
Simplified decay scheme of I-124 with a transition probability >3%, *maximal energy (β+: positron emission, γ: gamma emission, X: x-ray emission, ε: electron capture).

Due to the complex decay scheme of I-124 (see fig. 1, data taken from [6]) different challenges are to be met. Low energy photons cause strong geometry and vial dependency when measuring I-124 in dose calibrators. This fact requires additional efforts for correct activity quantification [7]. Furthermore, prompt gamma photons with an energy of 602.7 keV are emitted in cascade with about 50% of the positron emissions. Most of their energy spectrum lies within the standard energy window of a modern LSO PET-scanner (typically 425–650 keV) including those events scattered with a small angle; therefore additional and wrong (prompt) coincidences are detected. These coincidences are spread all over the field-of-view (FOV) of the PET-scanner and cause an approximately homogeneous background similar to the one from random coincidences. The prompt coincidences have to be removed, otherwise the image contrast is decreased causing overestimation of the activity in high density regions if attenuation correction is applied [8], [9]. To take these coincidences into account, a patented correction algorithm for non-standard isotopes was introduced by the manufacturer (named prompt-gamma correction (PGC)) and integrated within the reconstruction software [10].

To compare the quantification and image quality of I-124 measurements, the PET-nuclides F-18 and Ga-68 were used. F-18 has a much higher positron branching ratio of about 96% (I-124: 22.5%) and much lower positron emission energies (mean energies: I-124: 0.69 MeV and 0.97 MeV, F-18: 0.25 MeV). Therefore, differences between the I-124 and F-18 images cannot be assigned uniquely either to the lower branching ratio or to the higher positron energies. To overcome these problems the same measurements were repeated with Ga-68. Ga-68 has a similar branching ratio as F-18 (89.1%) and positron energies similar to I-124 (mean energy: 0.84 MeV). By comparing F-18, Ga-68 and I-124 differences in quantification and image quality can be better classified. In several studies PET-imaging with I-124 was studied [8], [11]–[19] comparing different parameters of I-124 PET-images with F-18, but not with Ga-68.

The aim of this study was to determine the quality of the quantification and image quality of I-124 PET-images by comparing the recovery coefficients (RC), the signal to noise ratios (SNR) and the relative contrasts to F-18 and Ga-68 in a series of phantom experiments. Furthermore, the influence of the offered PGC on the quantification and image quality is determined in conjunction with the sinograms.

## Materials and Methods

### Decay schemes of I-124

The decay scheme of I-124 is complex as it contains positron emission, electron capture and emission of x-rays and gamma-rays (see fig. 1). Due to the positron emission PET-imaging is possible; however, the branching ratio of positron emission with 22.5% is much lower than that for F-18 (96.7%). Longer measurements or higher activity concentrations are therefore necessary to get a similar image quality as compared to F-18. Furthermore, the emitted positrons have higher emission energies and therefore higher ranges than those emitted by F-18 resulting in a decreased spatial resolution of the PET-images. Consequently, the mean positron range differences between F-18 and Ga-68, I-124 are about 2–3 mm.

The major problem, however, are the gamma-photons with an energy of 602.7 keV as their energy lies within the standard energy window of a LSO PET-scanner (typically 425 keV–650 keV): most PET-scanners will also detect these photons as additional coincidences. The 602.7 keV photons have a high branching ratio of about 63%. In addition, these photons are emitted in cascade of nearly 50% of the positron emissions so the probability of such an additional (prompt) coincidence increases. As a consequence, the dead time of the system is increased and its count-rate performances significantly reduced [20].

### Activity Quantification

Apart from the 602.7 keV photons causing spurious coincidences, low energy photons with an energy of about 27 keV hamper the measurement of the absolute activity of I-124 within dose calibrators. Due to their low energy, these photons cause the measurement to be highly dependent on the sample geometry and the material of the vial containing the activity. This is mainly caused by the higher absorption and stopping power of the ionization chamber and the vial walls for low energetic photons. In order to overcome this issue, activity measurements were cross-calibrated against measurements of aliquots in a calibrated high-purity germanium detector [7].

To ensure that the dose calibrator measurements of I-124 remain independent on both the sample geometry and the material of the vial/syringe to be measured, a 1 mm cylindrical copper shielding was placed between the sample and the wall of the ionising chamber. The reading of the dose calibrator was cross-calibrated to an efficiency- and energy-calibrated high-purity germanium-detector, accounting for the copper insert.

### Phantoms

For the phantom measurements with F-18, Ga-68 and I-124 we used the body-mimicking emission PET-Phantom according to IEC 61675 (Manufacturer: PTW, Freiburg, Germany) with a background volume of 9.6 l. For the determination of the partial-volume effect (PV) and/or image quality, we used an insert supporting 6 hollow glass spheres with diameters of 10, 13, 17, 22, 28, and 37 mm and a wall thickness of about 1 mm. The centres of the spheres were laid in a plane parallel to the transaxial plane of the FOV. In order to simulate realistic hot, warm or cold lesions the spheres and eventually the background were filled with activity solution.

For the determination of the spatial resolution a cylindrical PET/SPECT phantom was used (volume: 5.6 l) containing two parallel line sources of 22 cm length with an interior diameter of 1 mm was used. The sources were filled with high specific activities of F-18, Ga-68 and I-124.

### Scanner

All studies were performed using a Biograph mCT® (Siemens AG Healthcare, Germany). This equipment provides a 64-slice CT, an axial field of view of 216 mm, a coincidence window of 4.1 ns, a timing resolution below 0.6 ns and an energy window of [435 : 650] keV. Time-of-flight reconstruction was not evaluated in this study. Measurements duration for F-18 and Ga-68 were set to 2 min as for the clinical routine. To compensate the lower branching ratio of I-124, longer scanning times were empirically determined by comparing the SNRs for different acquisition times to the SNRs of the F-18 and Ga-68 images. All measurements were performed in 3D mode.

### Reconstruction

All images were attenuation corrected and reconstructed with an Ordinary Poisson full-3D OSEM reconstruction algorithm with and without scatter correction. In addition, the influence of post-reconstruction filtering was studied by applying either no filtering or Gauss filters of different FWHM (2 mm and 5 mm). For I-124 each reconstruction was performed with and without PGC.

### Prompt-Gamma Compensation (PGC)

To avoid biased quantification and degradation of the image quality the prompt coincidences have to be accounted for and corrected [4], [5]. A prompt-gamma compensation (PGC) algorithm was made available from the manufacturer of the PET scanner (Siemens AG Healthcare, Germany). This approach calculates the distribution of the prompt coincidences using the convolution of a PGC kernel with attenuation corrected and for random coincidences corrected acquired data [5], [16]. The PGC kernel is determined analytically by means of a simplified model of the attenuation and the detection process of the prompt coincidences. Subsequently the model can be included in the forward scatter distribution calculated with a single scatter simulation algorithm [21]. The edges of the emission body shall then be identified (e.g. by means of matching CT) and all simulated contributions out of this limit be matched with a linear combination of the PGC and scatter distribution. The PGC contribution will then be rescaled and then subtracted from the measured emission [5].

To simplify the algorithm two assumptions are used. First, the attenuation of the prompt coincidences cannot be measured directly with transmission scans. Therefore, the attenuation of the prompt coincidences is estimated with the broad beam attenuation coefficient in water. Second, it is assumed that the difference of the distance between the two detectors from the place of the annihilation is similar [20].

### Image Quality and Quantification

To evaluate the image quality and quantification the relative contrast c, the isovolume RC, and the SNR of the different spheres in F-18, Ga-68, and I-124 images were calculated and compared:
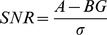


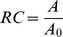


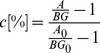



A is the measured activity of the spheres and BG the background activity in the PET-image with σ as the standard deviation of the background activity in the image. A_0_ and BG_0_ is the nominal activity of the spheres and background measured in dose calibrator.

Image analysis was performed with the Syngo.Via software VA10A (Siemens AG Healthcare, Erlangen, Germany). In order to determine the activity of the spheres, spherical volume of interests (VOIs) matching exactly the known volume of the spheres were placed into the images. Results such as mean, maximum and minimal specific activity/SUV of the VOI and the standard deviation of the activity in the corresponding VOI were reported. The background activity was determined placing an additional spherical VOI (containing the volume of the second biggest sphere) between the spheres assuming that the scatter contribution of each of the spheres in this area is equal.

### Measurements

To determine the image quality and quantification of I-124 images the PET/SPECT-phantom was filled with 5 different activity distributions. Two measurements were made without background activity and 2 different specific activities within the spheres. Furthermore 3 measurements with different sphere-to-background-ratios (S∶BG) were performed ensuring that the activities of the different isotopes were similar. The specific activities in the spheres varied between 26 kBq/ml and 570 kBq/ml, the background activities were approximately 2 kBq/ml. The S∶BG ratio was chosen between 10 and 20 (see table 1) as this study was basically aimed at improve and evaluating diagnostic quantitative imaging with I-124 for thyroid cancer patient dosimetry. Due to the specific uptake of iodine in iodine avid lesions S∶BG ratios of less than 10∶1 are very rarely observed in patients suffering from DTC. Another reason for using the specified S∶BG ratios was to provide better comparability with published data from Gregory et al. [11], Jentzen et al. [15], and Lubberink et al. [16].

**Table 1:** Specific activities and sphere-to-background-ratios used in PET-measurements.

		Measurement 1	Measurement 2	Measurement 3	Measurement 4	Measurement 5
	A_sphere_ [kBq/ml]	462	49.3	41.0	31.3	25.6
F-18	A_BG_ [kBq/ml]	0	0	2.2	2.2	2.1
	S∶BG	–	–	18.6∶1	14.2∶1	12.2∶1
	A_sphere_ [kBq/ml]	570	60.1	44.2	32.5	23.9
Ga-68	A_BG_ [kBq/ml]	0	0	2.2	2.2	2.4
	S∶BG	–	–	20.1∶1	14.8∶1	9.9∶1
	A_sphere_ [kBq/ml]	475	39.4	39.2	28.8	20.7
I-124	A_BG_ [kBq/ml]	0	0	1.9	1.9	2.1
	S∶BG	–	–	20.4∶1	15.3∶1	9.9∶1
	Purpose	SNR	RC; Comparison of Sinograms	RC and contrast; Comparison of Sinograms	RC and contrast; Comparison of Sinograms	RC and contrast; Comparison of Sinograms

(SNR: Signal-to-noise ratio, RC: recovery coefficient).

A comprehensive list of the activities used for the scans is given in table 1. The purpose of the different measurements is indicated in the last row of table 1.

## Results

### Uncertainties

The errors in the absolute quantification of the specific activity are determined by the errors due to the cross-calibration in the calibrated high-purity germanium detector against measurements of aliquots of about 2% and of the average error caused by the dose calibrator as given by the manufacturer of ±3% (with an additional dependency on the geometry and wall thickness of the vials used). As a conservative estimate a maximum error for the absolute activity quantification of 5% is assumed.

The second source of errors is the activity quantification by the PET scanner. An assessment of the errors relies on estimates due to the use of scatter correction, PGC and iterative reconstruction algorithms. In principle, the use of the standard deviation as given by the count-rates in the VOIs (e.g. used by Gregory et al. [11]) is not applicable as the activity determination close to the edge of the spheres suffers from partial volume effects. This is particularly problematic for the smallest spheres. An additional error between 0.2% (largest sphere) and 3% (smallest sphere) is caused by the manual placement of the VOIs. Summing up the contributions of the different sources of uncertainties the errors are estimated to be ±5% for the contrast measurements and ±10% for the RC measurements due to the additional uncertainties caused by the dose calibrator measurements.

### Spatial resolution

The transverse resolution (determined as the mean value of FWHM measurements) was 4.6 mm (F-18), 5.6 mm (Ga-68), 5.5 mm (I-124 with PGC) and 5.6 mm (I-124 without PGC). The corresponding values for the transaxial planes were 6.5 mm, 7.2 mm, 7.1 mm, and 6.9 mm, respectively. The differences in the transverse resolution are consistent with the mean positron range differences between F-18 and Ga-68, I-124.

### Signal-to-noise ratio

The SNRs are similar for F-18, Ga-68 (2 min acquisition time) and I-124 with PGC (10 min acquisition time). In order to get comparable count rates and standard deviations of the background the acquisition durations were adjusted according to the differing branching ratios. For I-124 without PGC the SNR is several orders of magnitude higher (this observation is discussed below).

### Image quality

**Figure 2:**
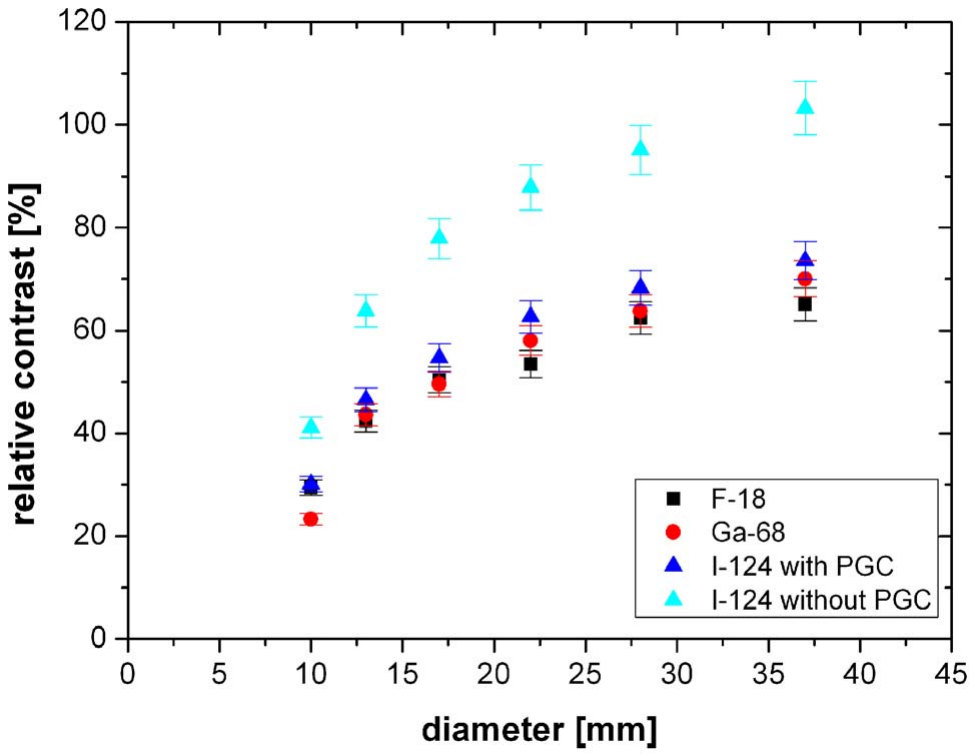
Relative contrast of F-18, Ga-68 and I-124 with and without PGC as a function of sphere diameter. Reconstruction iterative with scatter correction and a Gauss-filter with a FWHM of 2 mm and a S∶BG≈10∶1.

Using the relative contrast as parameter, images with different S∶BG ratios can be compared. In fig. 2 the relative contrast is plotted as a function of the sphere diameter for F-18, Ga-68 and I-124 with and without PGC. The highest contrast was achieved without post-reconstruction filtering and with a Gauss-filter with a FWHM of 2 mm, as shown in fig. 2. By using a Gauss-filter with a FWHM of 5 mm the contrast decreased (4%–8%). For smaller spheres the PV effect reduces the relative contrast. For the larger spheres the relative contrast reaches saturation.

The relative contrast of I-124 images with PGC amounted to74% for the largest sphere (see fig. 2). The relative contrasts for the largest sphere of F-18 (65%) and Ga-68 (70%) showed similar values. The difference between F-18 and I-124 with PGC decreased for the smaller spheres; the difference between Ga-68 and I-124 with PGC remained approximately constant (see fig. 2). I-124 images without PGC show higher values with a relative contrast of about 103% for the 25.4 ml sphere. The difference between I-124 images with PGC and I-124 images without PGC decreased from 29% for the largest sphere to 11% for the smaller spheres. However without the use of PGC the contrast for I-124 was overestimated.

### Quantification of I-124 images

**Figure 3:**
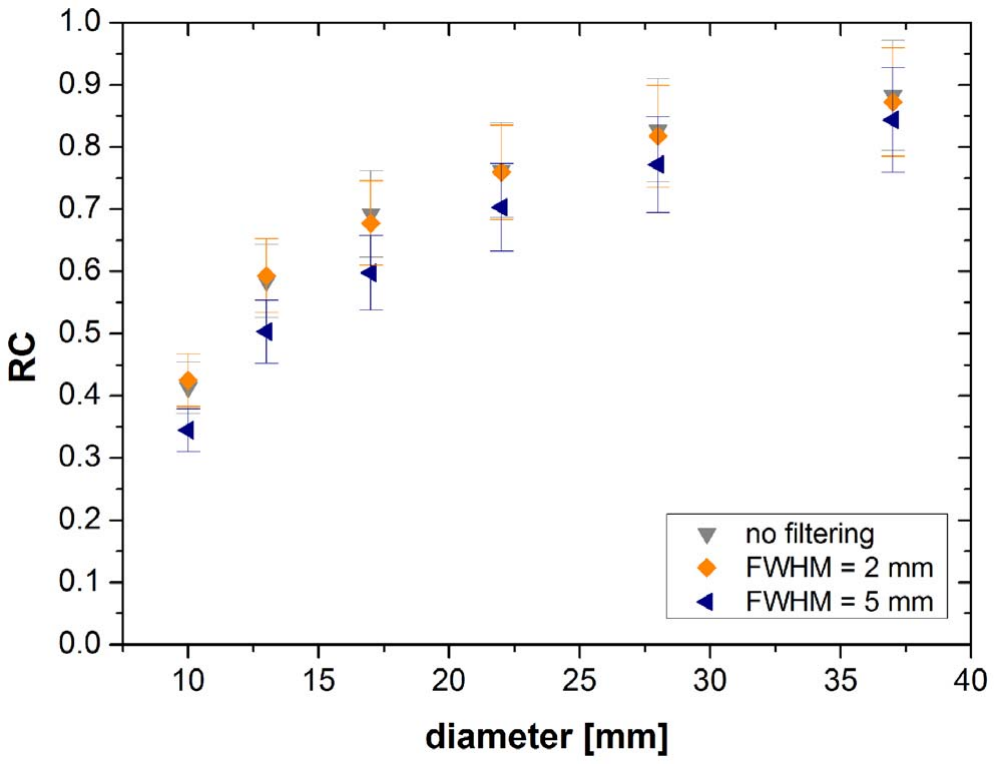
RC of I-124 with PGC depending on post-reconstruction filtering as a function of sphere diameter. Reconstruction iterative with scatter correction and a S∶BG = 9.9∶1.

In fig. 3 the isovolume RC is plotted as a function of the sphere diameter and of the post-reconstruction filtering. The isovolume RC calculated from images without post-processing filtering and with a Gauss-filter with a FWHM of 2 mm reached the highest RC while the RC for a Gauss-filter with a FWHM of 5 mm is slightly decreased (fig. 3). Furthermore the RC is also influenced by the PV effect with a decreasing RC for the smaller spheres.

**Figure 4:**
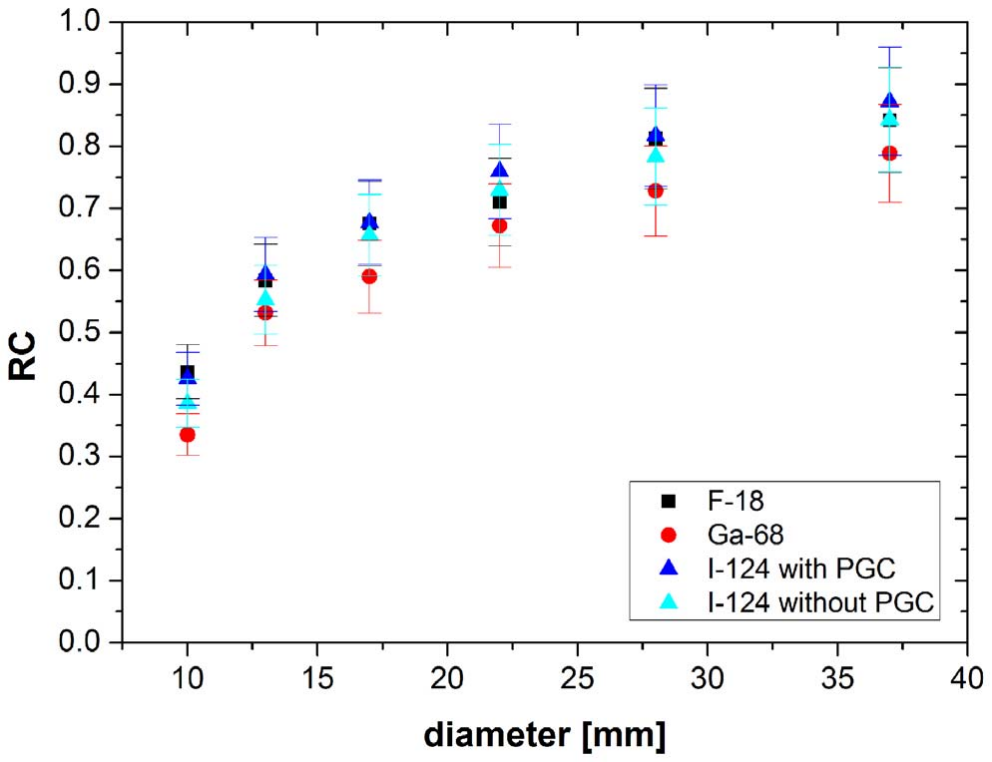
RC of F-18, Ga-18 and I-124 with and without PGC as a function of sphere diameter. Reconstruction iterative with scatter correction and a Gauss-filter with a FWHM of 2 mm and a S∶BG≈10∶1.

In fig. 4 the RC of F-18, Ga-18 and I-124 with and without PGC are drawn as a function of the sphere diameter using a S∶BG≈10∶1. The images were reconstructed with OSEM, scatter correction and a Gauss-filter with a 2 mm FWHM. I-124 images with PGC reached a RC of about 87% for the largest sphere. The RC of I-124 slightly decreased (3%–4% smaller) when PGC is not applied. The RC of F-18 showed similar values as I-124 with PGC (84% for the largest sphere). For the small spheres the difference between F-18 and I-124 with PGC were less than 1%. Comparing Ga-68 the RC reached lower values of about 79% for the largest sphere. The difference of 8% compared to I-124 with PGC did not change (fig. 4).

### Evaluation of the Prompt-Gamma Compensation with I-124

**Figure 5:**
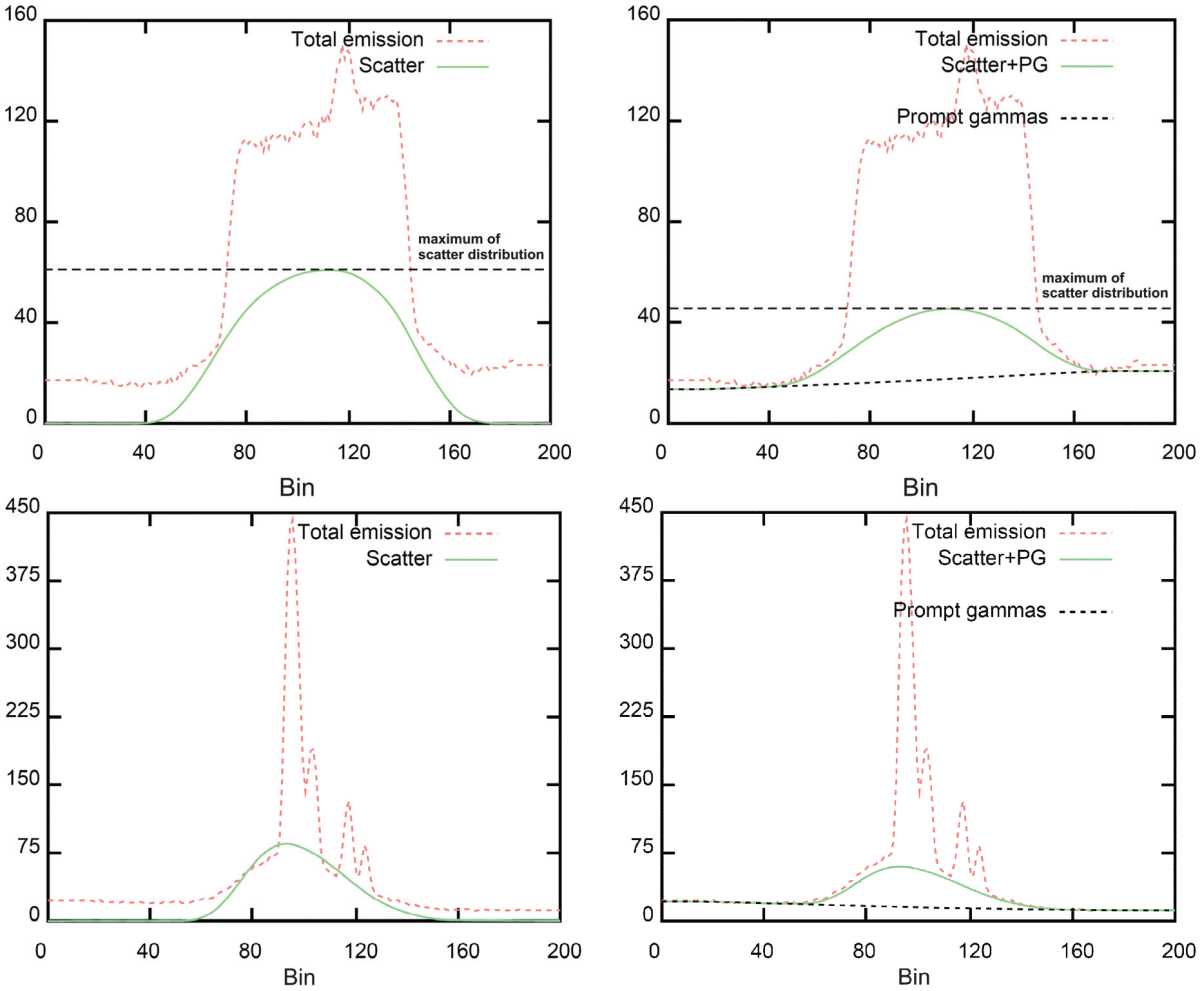
Sinograms of I-124 phantom images with PGC (right) and without PGC (left), top: with background activity, bottom: without background activity.

In fig. 5 the I-124-sinogram profiles with PGC (right) and without PGC (left) are given for selected planes (top: with background activity, bottom: without background activity). Regarding the I-124-sinogram profiles with PGC the scatter and PGC-distribution fits the measured emission using the PGC well. The simulated coincidences at the edge of the FOV get compensated and the shape of the scatter distribution matched the actual emission profile at the edge of the phantom. Regarding the sinograms with and without background activity the scatter correction and the PGC work comparably well (see fig. 5 right: top and bottom).

Without PGC the shape of the scatter distribution shows deviations to the measured total emission and a significant mismatch occurs (see fig. 5 left: top and bottom). The coincidence events at the edge of the FOV will not be considered properly by applying only the scatter correction algorithm. Furthermore, the shape of the scatter correction is not estimated correctly and does not match well the emission at the edge of the phantom; the slope of the scatter distribution is much steeper than the curve of the total emission. In addition, the calculated scatter distribution reaches higher maximal values (comparing fig. 5 top: left and right) which prevent direct scaling operations while correcting for scatter. Without background activity the scatter distribution surpasses partially the total emission in the middle of the FOV (see fig. 5 bottom left).

**Figure 6:**
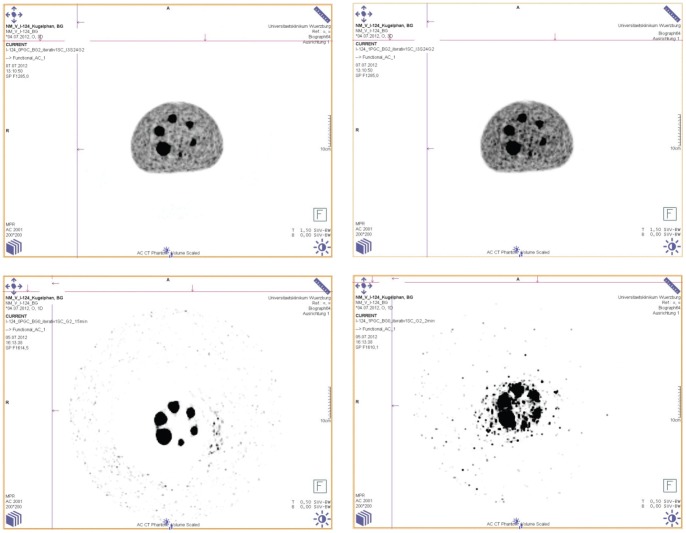
Transaxial views of I-124 phantom images with PGC (right) and without PGC (left), top: with background activity, bottom: without background activity.

For better illustrating this effect the corresponding I-124-transaxial views with PGC (right) and without PGC (left) are shown in fig. 6 for one selected plane (top: with background activity, max SUV: 1.5, bottom: without background activity, max SUV 0.5). For the images with background activity no changes in the background patterns are observed whereas the images containing no background activity show zero counts for the scans reconstructed without PGC (bottom left).

## Discussion

**I-124 images with PGC show similar relative contrast as F-18 and Ga-68 and thus a** comparable image quality. Thus, the contrast is similarly well rendered in images with standard PET-nuclides and I-124 with PGC. However, if the prompt coincidences correction algorithm is not applied the contrast and the SNR are unexpectedly high. This observation is most likely caused by an overcorrection of the background activity (voxel values lower than zero are set to zero, see also fig. 5 left panels) and a corresponding reduction of the standard deviation. Therefore, the background activity is not estimated correctly without the PGC algorithm.

If the PGC is not applied the calculated scatter distribution does not consider the background coincidences at the edge of the transaxial FOV. In this case, these coincidences will not be corrected and the activity in this area will be higher. Furthermore, the shape of the scatter distribution does not fit well the actual shape estimated from an emission profile. The distribution shows a different kurtosis and the simulated relative maximal values of the scatter profile are overestimated in comparison to those obtained with the PGC (see fig. 5 top). In the middle of the FOV the impact of scattering is overestimated without PGC and the resulting reconstructed activity in this area is therefore lower. Due to this overcorrection of the scatter correction without using the PGC the background activity is too low and, therefore, the contrast in the image too high. If there is no or low background activity at some places in the sinogram the scatter distribution reaches higher values than the measured emission (see fig. 5 bottom left). When applying the scatter correction these values are set to zero and the background activity and the noise in the background decrease artificially. This overcorrection during scatter correction has a large influence on small activities and small background activities. The effect of the overcorrection on the activity in lesions is much smaller but is not negligible.

When comparing the relative contrast of I-124 with PGC of the present work with data presented by Gregory et al [11], slight differences are observed. The measurements by Gregory et al. were made with a different PET system (Philips Gemini dual GS PET/CT) and were reconstructed with the algorithm RAMLA. For all spheres the published values of Gregory et al. are smaller than the measured contrast of this study (6%–17%). We suppose that the reconstruction algorithms and especially their transfer function and post-filtering properties usually impact RC, as well as the intrinsic resolution of the used scanner.

The RC of I-124 reaches similar values compared to F-18. Thus the quantification of I-124 images is as good as the quantification of F-18 and even better than that of Ga-68. Regarding the RC the influence of the PGC on the activity in lesions in images with I-124 seems to be low (differences 2%–4%, see fig. 4).

Furthermore, the post-reconstruction filtering and more generally the smoothness of images influence the RC. Without any filtering and with a Gauss-filter with a FWHM of 2 mm the RC show similar values (differences about 1%). Using a broader filter the local activity distributions spread over larger areas. Therefore, the activity in the volume of a sphere and its RC decrease (see fig. 3). For a sufficient quantification of the images either the post-reconstruction filtering should not be applied or a filter with a small FWHM should be used to increase slightly the SNR at the costs of a limited RC decrease.

Isovolume RCs for I-124 measurements were published by Jentzen et al [14]. A Biograph Emotion Duo (Siemens Erlangen) with a different intrinsic resolution was used; the images were reconstructed using an FORE+OSEM 2D algorithm. The published RC of I-124 is slightly lower than the RC for F-18 (about 14%). Comparing these values with the measured RC of this study the RC of Jentzen et al are smaller (difference 15%–23%).

In conclusion, the PGC in connection with scatter correction corrects prompt and scattered coincidences reliably. If the PGC is not applied the scatter correction possibly delivers erroneous background values in regions with low activities, which affects contrast and quantification in images. Hence, to get a reliable image quality and quantification the PGC should be used for reconstructing I-124 images.

## Conclusion

With respect to the relative contrast and the RC the I-124 image quality and quantification is comparable to that F-18 and Ga-68 images when applying the PGC and with a 5-fold longer acquisition time. If the PGC is not used the prompt coincidence level at the edge of the FOV will not be estimated properly while in the middle of the FOV too many coincidences get subtracted because of an overestimation of the scatter contribution. A conclusion of the experimental data presented in this work is that, if available, the PGC should be used for reconstructing I-124 images in order to get both reliable image quality and quantification.
